# Supplementary material for: Causal Relationship between Aging and Anorexia Nervosa: A White-Matter-Microstructure-Mediated Mendelian Randomization Analysis
Source: Biomedicines. 2024 Aug 16;12(8):1874. doi: 10.3390/biomedicines12081874 (PMC11351342; doi:10.3390/biomedicines12081874)
Supplement: Supplementary file 1 [file biomedicines-12-01874-s001.zip › Supplementary Figure/Figure S4/Singe SNP plot of MR analysis for TL to ubm-b-1871.pdf]

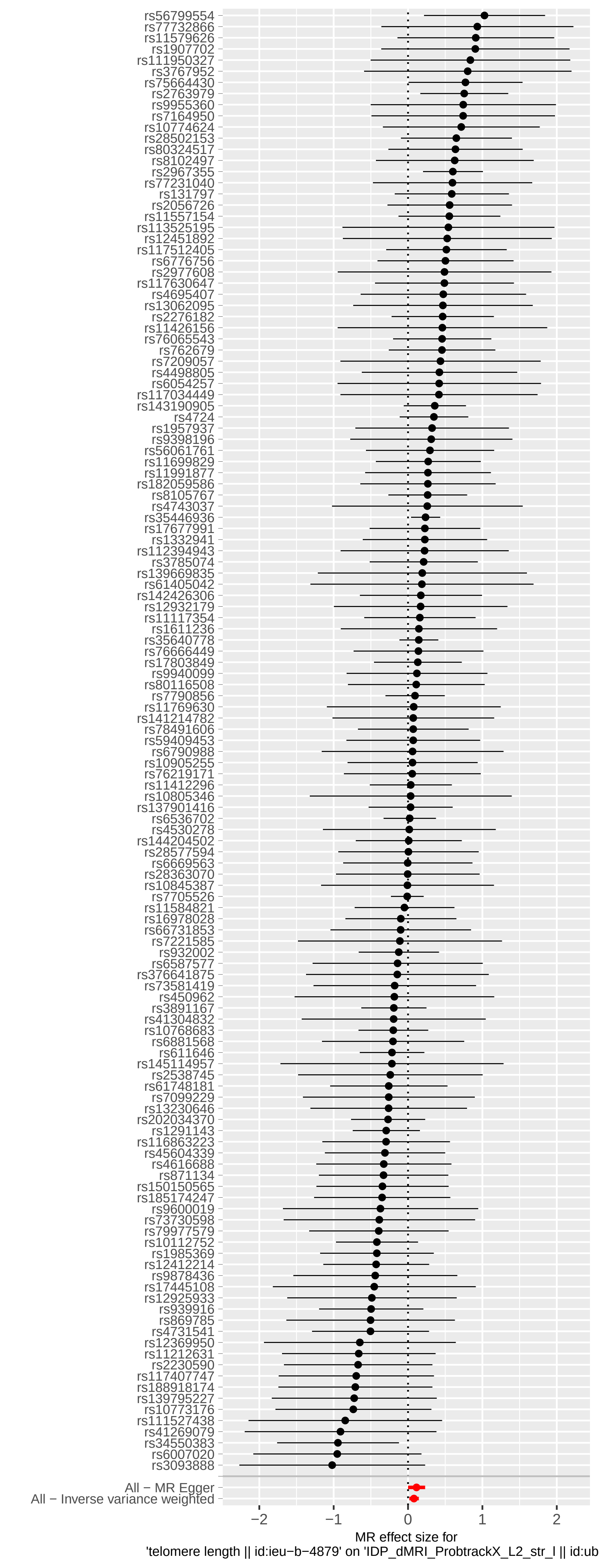

rs56799554
rs77732866
rs11579626
rs1907702
rs111950327
rs3767952
rs75664430
rs2763979
rs9955360
rs7164950
rs10774624
rs28502153
rs80324517
rs8102497
rs2967355
rs77231040
rs131797
rs2056726
rs11557154
rs113525195
rs12451892
rs117512405
rs6776756
rs2977608
rs117630647
rs4695407
rs13062095
rs2276182
rs11426156
rs76065543
rs762679
rs7209057
rs4498805
rs6054257
rs117034449
rs143190905
rs4724
rs1957937
rs9398196
rs56061761
rs11699829
rs11991877
rs182059586
rs8105767
rs4743037
rs35446936
rs17677991
rs1332941
rs112394943
rs3785074
rs139669835
rs61405042
rs142426306
rs12932179
rs11117354
rs1611236
rs35640778
rs76666449
rs17803849
rs9940099
rs80116508
rs7790856
rs11769630
rs141214782
rs78491606
rs59409453
rs6790988
rs10905255
rs76219171
rs11412296
rs10805346
rs137901416
rs6536702
rs4530278
rs144204502
rs28577594
rs6669563
rs28363070
rs10845387
rs7705526
rs11584821
rs16978028
rs66731853
rs7221585
rs932002
rs6587577
rs376641875
rs73581419
rs450962
rs3891167
rs41304832
rs10768683
rs6881568
rs611646
rs145114957
rs2538745
rs61748181
rs7099229
rs13230646
rs202034370
rs1291143
rs116863223
rs45604339
rs4616688
rs871134
rs150150565
rs185174247
rs9600019
rs73730598
rs79977579
rs10112752
rs1985369
rs12412214
rs9878436
rs17445108
rs12925933
rs939916
rs869785
rs4731541
rs12369950
rs11212631
rs2230590
rs117407747
rs188918174
rs139795227
rs10773176
rs111527438
rs41269079
rs34550383
rs6007020
rs3093888
All − MR Egger
All − Inverse variance weighted
−2
−1
0
1
2
MR effect size for
'telomere length || id:ieu−b−4879' on 'IDP_dMRI_ProbtrackX_L2_str_l || id:ub
